# Optimizing Probiotic Low‐Fat Yogurt: The Benefits of Incorporating Defatted Rice Bran for Enhanced Quality and Functionality

**DOI:** 10.1002/fsn3.4558

**Published:** 2024-11-04

**Authors:** Khalid A. Alsaleem, Mahmoud E. A. Hamouda

**Affiliations:** ^1^ Department of Food Science and Human Nutrition College of Agriculture and Food, Qassim University Buraydah Saudi Arabia; ^2^ Dairy Science Department, Faculty of Agriculture Assiut University Assiut Egypt

**Keywords:** defatted rice bran (DRB), functional properties, nutritional enhancement, probiotic low‐fat yogurt (LFY), rheological functions, sensory evaluation

## Abstract

This study investigated the effects of incorporating defatted rice bran (DRB) at different concentrations (0.5%, 1%, 1.5%, and 2%) on the quality, microbiological, and sensory characteristics of probiotic low‐fat yogurt (LFY) during a 21‐day storage period at 4°C. DRB is rich in dietary fiber, essential amino acids, vitamins, and bioactive compounds, and its addition aimed to enhance the nutritional and functional properties of LFY. LFY samples were evaluated for proximate composition, hardness, viscosity, syneresis, color characteristics, rheology, and sensory evaluation. Also, microbiological analysis includes total bacterial counts (TBC), *Lactobacillus delbrueckii* *ssp.* *bulgaricus*, *Streptococcus thermophilus*, *Bifidobacterium bifidum* counts. Results indicated that DRB addition significantly (*p* < 0.05) increased probiotic counts, especially at 1% and 1.5% concentrations, with these samples maintaining higher bacterial stability over the storage period. DRB‐added LFY also showed improved nutritional profiles, with increased TS, protein, ash, fiber, and antioxidant activity. Additionally, hardness, viscosity, and rheology (G′ and G″) values significantly (*p* < 0.05) increased with the addition of DRB, while syneresis significantly (*p* < 0.05) decreased. However, higher DRB concentrations negatively affected the color, with lightness decreasing and the browning index increasing. This also impacted sensory characteristics, resulting in lower scores for color, flavor, and overall acceptability, particularly in LFY with 2% DRB. To conclude, moderate DRB addition (up to 1.5%) optimally balances the enhancement of probiotic and nutritional properties with acceptable sensory quality offering a viable strategy for producing functional low‐fat yogurt.

## Graphical abstract

This study investigated the effects of incorporating defatted rice bran (DRB) at different concentrations (0.5%, 1%, 1.5%, and 2%) on the quality, microbiological, and sensory characteristics of probiotic low‐fat yoghurt (LFY) during a 21‐day storage period at 4°C. The addition of moderate levels of DRB (up to 1.5%) effectively balances the enhancement of probiotic and nutritional properties with acceptable sensory quality, presenting a viable strategy for producing functional low‐fat yogurt. A figure that best represents the scope of the paper. The image supplied should fit within the dimensions of 50 × 60 mm, and be fully legible at this size. Low fat yoghurt manufacturing.

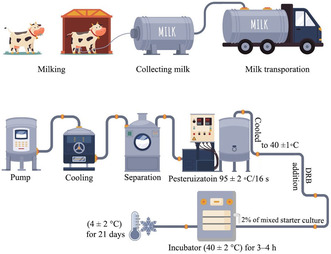

## 1 Introduction

As consumers become more health conscious, the demand for functional foods that offer added health benefits has significantly increased. Yoghurt, a staple in many diets, is particularly well‐suited to this trend, especially when fortified with probiotics live microorganisms that support gut health and boost the immune system (Sloan 2008). However, creating a low‐fat version of yogurt that retains its creamy texture and rich taste, while also enhancing its health benefits, presents several challenges. One of the biggest hurdles in developing low‐fat yogurt is maintaining its desirable texture and mouthfeel. Fat is integral to yogurt's sensory qualities, providing creaminess and viscosity that are often lost when fat content is reduced. Without adequate fat, yogurt can become thin and watery, which is generally less appealing to consumers. Various fat replacers, such as hydrocolloids and starches, have been used to address this issue, but they can sometimes introduce off‐flavors or textures that detract from the product's overall quality (Lukic et al. 2023; Publishing and Lucey 2004; Sandoval‐Castilla et al. 2004). Another major challenge is ensuring the survival and viability of probiotics in low‐fat yogurt. Probiotics need to remain alive throughout the product's shelf life to be effective. The reduced fat content can result in a less protective environment for these bacteria, making them more vulnerable to the acidic conditions of yogurt (Alsaleem and Hamouda 2024). Balancing the enhancement of probiotic viability with the preservation of the yogurt's sensory qualities requires careful formulation and process optimization (Ranadheera, Baines, and Adams 2010).

Defatted rice bran (DRB) is a by‐product of rice milling. Globally, a significant amount of rice bran, a by‐product of the rice milling process, is discarded. Approximately 60 million metric tons of rice bran are produced annually worldwide, but a large portion of this estimated at around 70% ends up as waste (Tan, Norhaizan, and Chan 2023). This high rate of disposal can be attributed to the rapid degradation of rice bran due to the action of lipase enzymes, which quickly rancidify its oil content, making it unsuitable for consumption or most industrial uses (Ju and Vali 2005). Efforts to stabilize rice bran through various treatments have been implemented to extend its shelf life and enhance its usability, aiming to reduce this substantial waste (Ju and Vali 2005). Defatting is a pivotal method used to stabilize rice bran and extend its shelf life by reducing its fat content, which is susceptible to rapid rancidity. This process typically involves the extraction of oil using solvents like hexane, or more environmentally friendly methods such as supercritical CO_2_ extraction (Yılmaz Tuncel 2023). By removing a significant portion of the oil, the rice bran becomes less prone to oxidative degradation, which enhances its nutritional stability and preserves its valuable components, such as proteins, vitamins, and dietary fibers (Nagendra Prasad et al. 2011). DRB can then be used in various applications, including the production of dietary supplements, functional foods, and as a feed ingredient, thus reducing waste and promoting sustainable usage of this by‐product (Spaggiari et al. 2021).

DRB offers a promising solution to low‐fat yogurt challenges. DRB is packed with dietary fiber, essential amino acids, and bioactive compounds like antioxidants, vitamins, and minerals. Its high fiber content can help improve the texture and mouthfeel of low‐fat yogurt, potentially making up for the absence of fat. Moreover, the bioactive components in DRB can enhance the nutritional profile of the yogurt, aligning with the trend toward more health‐conscious eating (Fabian and Ju 2011; Shen et al. 2009).

Incorporating DRB into yogurt may show potential benefits, such as improved viscosity and water‐holding capacity, which are crucial for achieving a desirable texture. Additionally, DRB's prebiotic properties can promote the growth and viability of probiotic bacteria, potentially creating a synergistic effect that enhances the health benefits of the yogurt (Antunes et al. 2023). Despite the promising attributes of DRB, there are still significant gaps in the existing research that need to be addressed. Firstly, while many studies have explored the use of various fibers and by‐products in yogurt, there is limited research specifically on the application of DRB in low‐fat yogurt formulations. Most existing studies have focused on the effects of DRB in bakery products, leaving a gap in our understanding of its impact on low‐fat dairy products (Tan, Norhaizan, and Chan 2023). Additionally, the interaction between DRB and probiotic bacteria within the yogurt matrix is not well understood. Although DRB is believed to enhance probiotic viability, the mechanisms behind this interaction require further investigation (Prado et al. 2008). Detailed studies are needed to examine how the fiber and bioactive compounds in DRB affect the survival, growth, and activity of different probiotic strains in low‐fat yogurt throughout its shelf life. Another gap pertains to consumer acceptance and sensory evaluation of low‐fat yogurt enriched with DRB. Sensory attributes such as taste, texture, and overall acceptability are critical for the success of functional food products in the market (Yin and Yin 2024).

Adding DRB to low‐fat yogurt may achieve significant potential for creating a functional dairy product that meets health and sensory demands. Therefore, our study aimed to investigate the effects of incorporating DRB at four different percentages into the manufacturing process of probiotic low‐fat yoghurt.

## 2 Materials and Methods

### 2.1 Experimental Design

In this study, we investigated the impact of adding DRB at four different percentages on the quality and characteristics of low‐fat yogurt. Our experimental design aimed to evaluate the effects of different DRB concentrations (treatments) over 21 days of storage at 4°C (Storage) and their interactions (treatment × storage). The study included a control group with no DRB addition and four treatment groups: T1 with 0.5% DRB, T2 with 1% DRB, T3 with 1.5% DRB, and T4 with 2% DRB. The yogurt samples were prepared and stored for 21 days, with analyses conducted at four storage intervals: 0, 7, 14, and 21 days at 4°C.

### 2.2 Preparing DRB

Rice bran (RB) was initially sieved to remove husk, grits, and other major components. Subsequently, the RB was defatted following the method described by Wang, Murphy, and Johnson (1999). The defatting process involved mixing RB with hexane in a 1:6 (w/v) ratio of bran to hexane, and stirring the mixture in a shaker (Cinetic, CT‐712RNT, Brazil) at room temperature for 6 h. After removing the hexane, the wet defatted bran was left in an open container for 18 h to allow the remaining hexane to evaporate. The dried DRB was then placed on aluminum plates and heated in an oven at 45°C–50°C for 30 min. Finally, the bran was sieved using a 100‐mesh sieve (0.150 mm) to obtain the final product.

### 2.3 Low‐Fat Yoghurt Manufacturing

**FIGURE 1:**
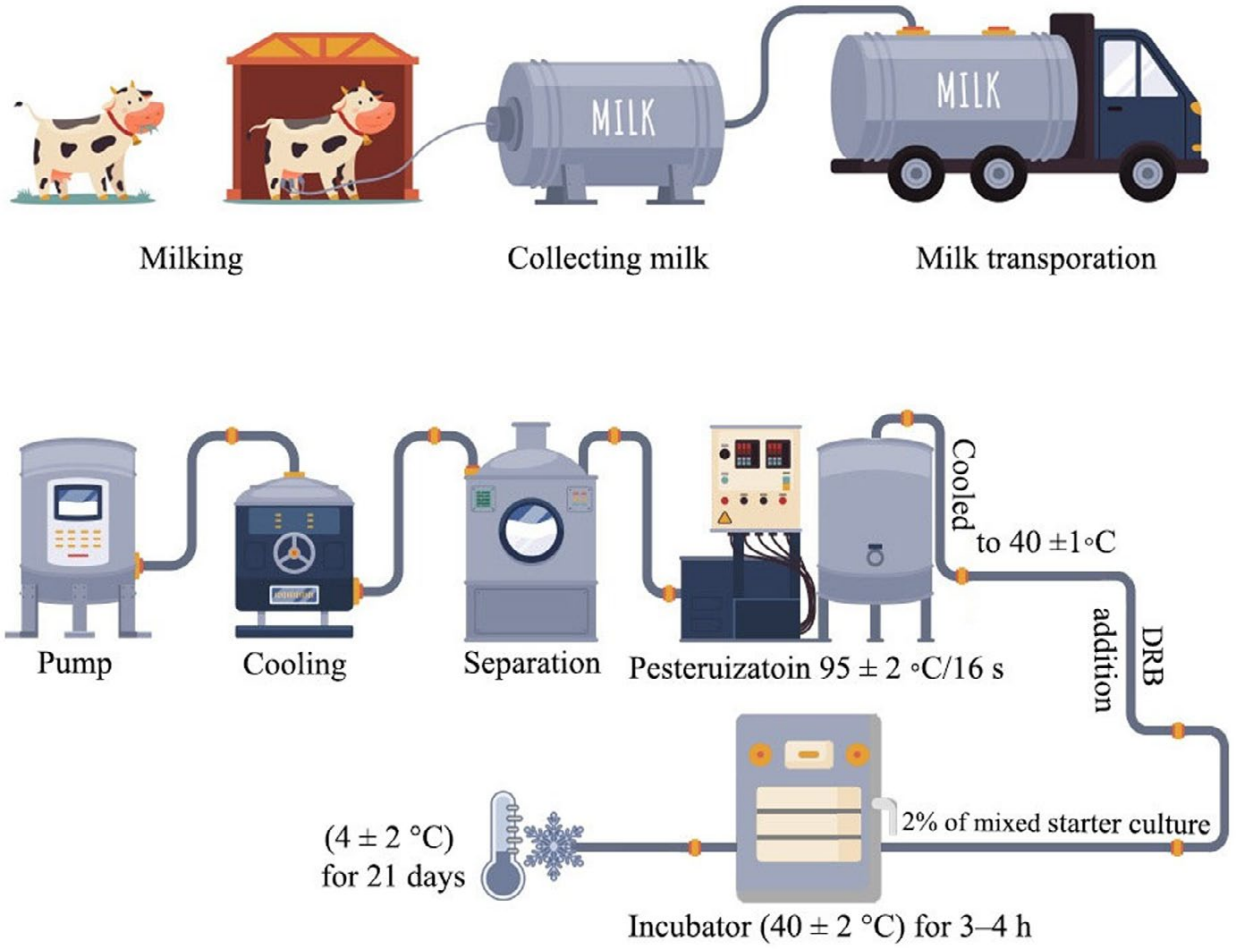
Low‐fat yogurt manufacturing.

Low‐fat yogurt manufacturing was done as described by Alsaleem and Hamouda (2024) with some modifications as shown in Figure 1. Fresh cow's milk (Farm of Faculty of Agriculture, Assiut University, Egypt) was separated at 4°C to produce SBM. The pasteurized skimmed cow milk was heated to 95°C± 2°C/16 s, cooled to 40°C± 1°C, and then divided into five treatment lots, as shown in Table 1. DRB was added to the SBM. Then, 2% of mixed starter culture from *Lactobacillus dlebreuckii* ssp., *L. bulgaricus*, *Streptococcus thermophilus*, and *Bifidobacterium bifidum* (Egyptian Microbial Culture Collection: EMCC, Cairo MIRCEN, Faculty of Agriculture, Ain Shams University, Cairo, Egypt) (1:1:1) was added to SBM. Prepared yogurt samples were kept at incubator temperature (40°C± 2°C) for 3–4 h until the pH reached 4.6; then, the samples were kept at refrigeration temperature (4°C± 2°C) for 21 days.

**TABLE 1:** Defatted rice bran (DRB) addition levels in low‐fat yoghurt manufacturing.

Treatments	Percentage of DRB %
Control	—
LFY 1	0.5
LFY2	1
LFY 3	1.5
LFY 4	2

### 2.4 Proximate Composition

All chemicals used in this experiment were sourced from BDH (Mumbai, India), Sigma (New Delhi, India), and Prolabo Chemicals (Mumbai, India). The pH of the yogurt samples was determined using a digital pH meter, following the method outlined in (AOAC Official Method 2015.01 Heavy Metals in Food, n.d.) The low‐fat yogurt (LFY) samples were analyzed for total protein (TP), total solids (TS), and ash content, fiber content, total phenolics, and antioxidant activity. TS content was determined using a forced‐draft oven, according to (AOAC Official Method 2000) Ash content was estimated using a muffle furnace at 550°C, based on the method described by Ling (1963). TP was determined using the Kjeldahl method (AOAC 2000; method 990.20; 33.2.44) employing a conversion factor of 6.38. Fiber content was determined according to methods of A.O.A.C. (1995). Total phenolics were assessed as described by the Folin–Ciocalteu spectrophotometric method (Singleton and Rossi 1965), and as standard gallic acid was used. The results were expressed as milligrams of gallic acid equivalents (GAE) per 100 g of yogurt sample on dry weight basis. Antioxidant activity (DPPH) assay was carried out according to the method described by Lee et al. (1998). The chemical composition was monitored at four storage intervals: 0, 7, 14, and 21 days.

### 2.5 Textural Analysis

Hardness was conducted using a texture analyzer (Stable Micro Systems, Godalming, Surrey, UK) equipped with a back‐extrusion plate probe (Probe P‐75, 75 mm diameter), as described by Gharibzahedi et al. (2014). The Texture Exponent 32 software was used to operate the texture analyzer. The analysis involved compressing the yogurt samples within their containers at a test speed of 0.5 mm/s, with a holding time of 2 s and a data acquisition rate of 200 cps. The parameters measured included firmness, consistency, cohesiveness, and adhesiveness, providing a comprehensive textural profile. The textural analysis was performed at 0, 7, 14, and 21 days.

### 2.6 Viscosity Determination

The viscosity of the yogurt was measured using a Brookfield LVDVE‐230 viscometer (Cole‐Parmer Scientific Experts, East Bunker Ct, Vernon Hills, IL, USA). Prior to measurement, the yogurt samples were stirred for 40 s. The viscosity was then determined at a temperature of 15°C± 1°C using spindle number 4 at 10 rpm. The results were recorded in centipoises (CPS) units. Viscosity measurements were conducted at 0, 7, 14, and 21 days.

### 2.7 Syneresis

Syneresis was measured following the method described by Keogh and O'kennedy (1998). A 30‐g sample of low‐fat yogurt (LFY) was centrifuged at 230 g for 15 min at 4°C using a ST Plus series centrifuge (Thermo Fisher, Bremen, Germany). After centrifugation, the clear supernatant was weighed and recorded as a percentage of syneresis. Syneresis was monitored at four storage intervals: 0, 7, 14, and 21 days.

### 2.8 Color Characteristics

The color of LFY samples was determined according to the method described by Park (2009) using a Hunter colorimeter with an optical sensor (Momcolor Inc., Columbus, OH, USA) based on the CIE L*, a*, and b* color space. The L* value measures lightness, ranging from black (0) to white (100). The a* parameter describes the red‐green spectrum, with positive values indicating redness and negative values indicating greenness. The b* parameter describes the yellow‐blue spectrum, with positive values indicating yellowness and negative values indicating blueness. The browning index (BI) was calculated based on the values of the L*, a*, and b* parameters according to Alsaleem et al. (2023) using the Equation (1) as follows:

$$\mathrm{BI}=\left[100\left(X-0.31\right)\right]/0.172$$

(1) $$\text{where}\;x=\left(a^{*}+1.75L^{*}\right)/\left(5.645L^{*}+a^{*}-3.012b^{*}\right)$$

### 2.9 Rheological Characteristics

The rheological characteristics of the low‐fat yogurt (LFY) were assessed using an Anton Paar rheometer (Anton Paar, Graz, Austria) equipped with a cylinder cup (42.01 mm inner diameter) and bob (38.69 mm outer diameter, 60.02 mm length effect, 143.8 mm active length, and 72.50 mm positioning length) as described by Wu et al. (2023) with some modifications. Prior to testing, the temperature of the 60‐mL samples was maintained at 25°C. The storage modulus (G′), which reflects the elastic or “solid‐like” behavior, and the loss modulus (G″), indicating the viscous or “liquid‐like” behavior, were measured at 25°C. The tests were conducted at an applied shear strain of 0.5% and an angular frequency range of 0–115 rad/s, with data points collected at a rate of 5 rad/s. Rheological characteristics are determined at only fresh time.

### 2.10 Microbiological Analysis

Under aseptic conditions, 1 g of low‐fat yogurt (LFY) samples was weighed and transferred into a sterilized jar. Next, 9 mL of sterile phosphate buffer was added and thoroughly mixed to achieve a 1:10 dilution, which was used to prepare subsequent dilutions (Wehr and Frank 2004). The total bacterial count (TBC) was determined by plating in duplicate on nutrient agar medium and enumerating using the standard plate count technique (Wehr and Frank 2004). The plates were incubated at 32°C for 48–72 h before microbial enumeration. *Lactobacillus delbrueckii ssp. bulgaricus* counts were determined using MRS agar medium (De Man, Rogosa, and Sharpe 1960) with plates incubated at 37°C for 48 h under anaerobic conditions. *Streptococcus thermophilu*s counts were enumerated using M17 agar medium (Wehr and Frank 2004). *Bifidobacterium* spp. counts were enumerated according to Brewer (1940) using modified MRS agar medium (m‐MRS), supplemented with 0.05% L‐cysteine HCl and 0.3% lithium chloride. These plates were also incubated anaerobically at 37°C for 48 h. Small white colonies were counted as colony‐forming units (CFU). Microbiological analyses of LFY samples from different treatments were conducted at four storage intervals: 0, 7, 14, and 21 days.

### 2.11 Sensory Evaluation

The sensory characteristics of the low‐fat yogurt (LFY) samples were assessed by a panel of 10–15 trained panelists from the Dairy Science Department at Assiut University. The evaluation followed the method described by Hamdy et al. (2020), with some modifications. The samples were rated on color and appearance (15 points), flavor (50 points), and body and texture (35 points), for a total of 100 points. The organoleptic characteristics were evaluated weekly when the samples were fresh, and subsequently at 14 and 21 days of storage.

### 2.12 Statistical Analysis

Data were analyzed using Costat 6.303 software (Gomez and Gomez 1984). Analysis of variance (ANOVA) was performed for each variable using a general linear model (GLM) to study the effects of treatments and storage time on the characteristics of low‐fat yogurt (LFY) made with DRB. Mean separation was conducted using the least significant difference (LSD) test when significant differences were detected at *p* ≤ 0.05.

## 3 Results and Discussion

### 3.1 Proximate Chemical Composition

Table 2 shows that the various concentrations of DRB (treatments), the different storage periods (storage) at 4°C, and the interaction between them (treatments × storage) significantly (*p* < 0.05) affected the pH of LFY samples supplemented with DRB. Moreover, the treatments had a significant (*p* < 0.05) impact on TS, ash, protein, fiber, and antioxidant activity percentages. Conversely, the storage and the interaction between the treatments and storage did not significantly (*p* > 0.05) influence these parameters.

**TABLE 2:** Proximate chemical composition (*n* = 3) of LFY samples supplemented with different concentrations of DRB during 21‐day storage at 4°C.

Treatments^a^	Storage^b^	pH	TS%	Ash%	Protein%	Fiber%	Antioxidant activity %
Control	0	4.60 ± 0.00	10.25 ± 0.21	0.77 ± 0.00	4.56 ± 0.21	0.00 ± 0.00	21.25 ± 0.36
	7	4.45 ± 0.02	10.25 ± 0.09	0.75 ± 0.00	4.25 ± 0.09	0.11 ± 0.00	22.32 ± 0.40
	14	4.39 ± 0.00	10.11 ± 0.15	0.78 ± 0.00	4.48 ± 0.15	0.13 ± 0.00	21.41 ± 0.52
	21	4.01 ± 0.00	10.12 ± 0.54	0.77 ± 0.00	4.55 ± 0.12	0.11 ± 0.00	19.25 ± 0.22
LFY 1	0	4.60 ± 0.02	11.21 ± 0.14	0.86 ± 0.00	4.57 ± 0.07	0.15 ± 0.00	22.65 ± 0.38
	7	4.43 ± 0.00	11.34 ± 0.11	0.85 ± 0.00	4.64 ± 0.11	0.15 ± 0.00	23.58 ± 0.11
	14	4.31 ± 0.00	11.39 ± 0.05	0.82 ± 0.00	4.58 ± 0.02	0.14 ± 0.00	23.68 ± 0.52
	21	3.89 ± 0.01	11.35 ± 0.31	0.87 ± 0.00	4.62 ± 0.01	0.15 ± 0.00	22.39 ± 0.25
LFY 2	0	4.60 ± 0.03	11.91 ± 0.22	0.92 ± 0.00	4.82 ± 0.11	0.23 ± 0.00	27.56 ± 0.29
	7	4.40 ± 0.00	11.85 ± 0.09	0.90 ± 0.00	4.78 ± 0.00	0.23 ± 0.00	28.95 ± 0.41
	14	4.22 ± 0.02	11.91 ± 0.08	0.93 ± 0.00	4.85 ± 0.00	0.23 ± 0.00	26.84 ± 0.19
	21	3.55 ± 0.01	11.95 ± 0.16	0.91 ± 0.00	4.81 ± 0.00	0.21 ± 0.00	29.32 ± 0.28
LFY 3	0	4.60 ± 0.00	12.25 ± 0.07	0.95 ± 0.01	4.96 ± 0.00	0.25 ± 0.00	33.65 ± 0.45
	7	4.35 ± 0.00	12.31 ± 0.12	0.97 ± 0.02	4.95 ± 0.00	0.25 ± 0.00	33.69 ± 0.18
	14	4.12 ± 0.02	12.02 ± 0.22	0.92 ± 0.00	4.89 ± 0.00	0.27 ± 0.00	34.25 ± 0.21
	21	3.38 ± 0.00	12.17 ± 0.25	0.95 ± 0.00	4.95 ± 0.00	0.25 ± 0.00	32.65 ± 0.17
LFY 4	0	4.60 ± 0.01	12.54 ± 0.09	1.07 ± 0.02	5.21 ± 0.00	0.31 ± 0.00	35.98 ± 0.61
	7	4.21 ± 0.02	12.84 ± 0.04	1.11 ± 0.10	5.15 ± 0.00	0.29 ± 0.00	39.32 ± 0.25
	14	3.61 ± 0.01	12.25 ± 0.13	1.05 ± 0.01	5.12 ± 0.00	0.34 ± 0.00	36.56 ± 0.25
	21	3.02 ± 0.02	12.39 ± 0.12	1.09 ± 0.02	5.29 ± 0.00	0.30 ± 0.00	37.58 ± 0.12
Treatments		(< 0.05)*	(< 0.05)*	(< 0.05)*	(< 0.05)*	(< 0.05)*	(< 0.05)*
Storage		(< 0.05)*	NS	NS	NS	NS	NS
(Treatments × Storage)		(< 0.05)*	NS	NS	NS	NS	ND

^a^ Treatments: control sample (low‐fat yogurt made without the addition of DRB), LFY1 = (low‐fat yogurt made with the addition of 0.50% DRB), LFY2 = (low‐fat yogurt made with the addition of 1% DRB), LFY3 = (low‐fat yogurt made with the addition of 1.5% DRB), and LFY4 = (low‐fat yogurt made with the addition of 2% DRB).

^b^ Storage: 0, 7, 14, and 21 days of storage at 4°C.

* Statistically significant at *p* < 0.05.

The results in Table 2 showed the impact of DRB on the proximate chemical composition of LFY over a 21‐day storage period. Control sample recorded the highest Ph and the lowest amount of TS, protein, and ash as compared to DRB‐added samples. Notably, as the concentration of DRB increases from 0.50% to 2.00%, a distinct increase in the acidity (decrease in pH), ash, TS, and protein was observed. The decrease in pH observed with increasing additions of DRB in yogurt could be attributed to enhanced fermentation activity. DRB is rich in nutrients that provide additional substrates for the probiotic bacteria in yogurt (Demirci et al. 2017). These bacteria metabolize sugars and other components in DRB to produce lactic acid, which lowers the yogurt's pH. Furthermore, the presence of vitamins, minerals, and possibly other growth factors in DRB might stimulate the metabolic activities of these cultures, thereby accelerating the acid production process. As a result, higher concentrations of DRB lead to increased lactic acid production and a consequent reduction in pH during storage time.

Increasing occurred in TS, ash, protein, and fiber by DRB addition and also by increasing the addition rate is expected because DRB contains almost 99% TS, approximately 18.97% protein, 10.5% ash, and 27% fiber. The observed increases in total solids (TS), ash, protein, and fiber content in LFY samples supplemented with DRB can be directly attributed to the compositional characteristics of DRB. DRB had a composition of nearly 99% total solids, which substantially increased the TS content in the LFY. Additionally, DRB contains high levels of protein (18.97%), ash (10.5%), and fiber (27%) (El‐Gammal 2017; Shaheen, Ahmad, and Anjum 2012) which directly contribute to increasing these specific nutritional metrics in the LFY formulation.

Foods with high antioxidant content are essential for maintaining oxidative balance in the body, supporting immune function, and slowing the aging process. Moreover, antioxidants can help preserve food quality by preventing the oxidation of fats and oils, thereby extending shelf life and retaining nutritional value (Carlsen et al. 2010). Results of antioxidant activity % also shown in Table 2 and ranged from 19.25% for control at 21 days of storage to 39.32% for LFY4 at 7 days of storage. The addition of DRB to LFY led to a significant (*p* < 0.05) which could be due to the natural composition of DRB, which is rich in bioactive compounds such as tocopherols, tocotrienols, and oryzanol. These compounds are known for their potent antioxidant properties, capable of scavenging free radicals and reducing oxidative stress in the body (Das et al. 2017). Also, Šmídová and Rysová (2022) studied the impact of adding DRB to gluten‐free products and he stated that addition of DRB can lead to a significant impact on increasing the antioxidant activity in bakery products and he explained this increase due to that DRB contains higher percentages of total phenolic components which act like antioxidant activity. On the other hand, Demirci et al. (2017) mentioned that increasing rice bran addition in yogurt from 1% to 3% led to a significant increase in antioxidant activity. Similar results obtained by Abbas, Hussien, and Ibrahim (2016).

The increases in these parameters with higher addition rates of DRB indicate the nutrient‐rich profile of DRB, effectively fortifying the yoghurt with essential nutrients and dietary fiber. This enhancement aligns with the goals of functional food development, where ingredient additions are tailored to improve nutritional value and health benefits.

### 3.2 Hardness

The hardness of yogurt is a critical textural attribute that impacts consumer preference and perception of quality. It is determined by the yogurt's fermentation process, protein content, and the presence of additives or thickeners, which can influence its firmness and cohesiveness (Lesme et al. 2020).

Table 3 shows that the various concentrations of DRB (treatments), the different storage periods (storage) at 4°C, and the interaction between them (treatments × storage) significantly (*p* < 0.05) affected hardness values of LFY during 21 days of storage time.

**TABLE 3:** Physical and color characteristics (*n* = 3) of LFY samples supplemented with different concentrations of DRB during 21‐day storage at 4°C.

Treatments^a^	Storage^b^	Hardness (g)	Viscosity (cp)	Syneresis%	*L* *	*b* *	*a* *	BI
Control	0	0.56 ± 0.03	1654.80 ± 2.03	39.00 ± 0.50	62.55 ± 0.25	22.26 ± 0.36	12.52 ± 0.22	57.30 ± 0.89
	7	0.51 ± 0.07	1685.21 ± 1.38	41.00 ± 0.00	63.52 ± 0.05	22.58 ± 0.22	13.25 ± 0.28	57.85 ± 0.96
	14	0.48 ± 0.01	1725.23 ± 3.14	45.00 ± 0.00	61.25 ± 0.21	23.51 ± 0.36	11.98 ± 0.05	61.24 ± 1.52
	21	0.46 ± 0.02	1805.25 ± 2.75	52.00 ± 0.00	62.28 ± 0.30	22.56 ± 0.25	12.75 ± 0.42	58.59 ± 0.82
LFY 1	0	0.61 ± 0.05	2021.32 ± 5.25	36.00 ± 0.00	59.08 ± 0.02	24.69 ± 0.09	15.69 ± 0.51	71.68 ± 1.36
	7	0.60 ± 0.04	2089.05 ± 4.11	37.00 ± 0.00	60.23 ± 0.35	24.58 ± 0.34	14.95 ± 0.12	68.86 ± 2.31
	14	0.55 ± 0.08	2125.55 ± 2.75	40.00 ± 0.00	58.36 ± 0.15	24.95 ± 0.05	15.26 ± 0.09	72.96 ± 2.09
	21	0.52 ± 0.01	2148.45 ± 3.58	47.00 ± 0.00	60.01 ± 0.52	24.98 ± 0.52	15.01 ± 0.31	70.31 ± 1.98
LFY 2	0	0.69 ± 0.01	2169.21 ± 2.98	34.00 ± 0.00	54.21 ± 0.09	27.69 ± 0.07	17.56 ± 0.02	91.92 ± 1.86
	7	0.65 ± 0.04	2189.25 ± 6.98	36.00 ± 1.00	55.36 ± 0.62	27.98 ± 1.01	16.08 ± 1.02	88.58 ± 2.39
	14	0.61 ± 0.07	2198.98 ± 2.11	38.00 ± 0.00	56.85 ± 0.39	28.91 ± 0.36	16.58 ± 0.31	89.24 ± 3.58
	21	0.58 ± 0.03	2235.55 ± 7.25	44.00 ± 0.00	52.86 ± 0.29	27.09 ± 0.21	17.09 ± 0.06	92.20 ± 3.66
LFY 3	0	0.75 ± 0.02	2354.21 ± 8.56	31.00 ± 0.00	49.36 ± 0.71	33.20 ± 0.29	18.63 ± 0.53	129.26 ± 5.25
	7	0.73 ± 0.12	2398.25 ± 9.11	33.00 ± 0.50	50.33 ± 0.53	33.87 ± 0.69	18.75 ± 0.43	129.00 ± 5.02
	14	0.69 ± 0.05	2421.97 ± 3.55	36.00 ± 0.00	45.25 ± 0.15	32.28 ± 0.78	19.05 ± 0.49	141.98 ± 6.89
	21	0.65 ± 0.01	2452.65 ± 1.98	41.00 ± 0.00	48.09 ± 0.37	34.29 ± 0.42	18.55 ± 0.29	139.54 ± 8.95
LFY 4	0	0.81 ± 0.05	2532.28 ± 5.69	25.00 ± 1.00	43.21 ± 0.59	38.23 ± 1.05	23.35 ± 0.54	198.01 ± 9.65
	7	0.81 ± 0.09	2597.09 ± 7.25	39.00 ± 0.00	41.56 ± 0.04	37.25 ± 0.28	23.25 ± 0.15	202.87 ± 8.25
	14	0.77 ± 0.01	2619.85 ± 5.22	31.00 ± 0.00	39.25 ± 0.20	39.12 ± 0.22	24.08 ± 0.86	241.74 ± 6.25
	21	0.71 ± 0.02	2675.05 ± 4.05	33.00 ± 0.00	42.36 ± 0.65	36.09 ± 0.61	22.58 ± 0.66	187.26 ± 7.61
Treatments		(< 0.05)*	(< 0.05)*	(< 0.05)*	(< 0.05)*	(< 0.05)*	(< 0.05)*	(< 0.05)*
Storage		(< 0.05)*	(< 0.05)*	(< 0.05)*	NS	NS	NS	NS
(Treatments × Storage)		(< 0.05)*	(< 0.05)*	(< 0.05)*	NS	NS	NS	NS

^a^ Treatments: control sample (low‐fat yogurt made without the addition of DRB), LFY1 = (low‐fat yogurt made with the addition of 0.50% DRB), LFY2 = (low‐fat yogurt made with the addition of 1% DRB), LFY3 = (low‐fat yogurt made with the addition of 1.5% DRB), and LFY4 = (low‐fat yogurt made with the addition of 2% DRB).

^b^ Storage: 0, 7, 14, and 21 days of storage at 4°C.

* Statistically significant at *p* < 0.05.

Control recorded the lowest values of hardness as compared to DRB‐added LFY samples. The addition of DRB significantly (*p* < 0.05) enhanced the hardness values which was mainly due to the high fiber content of DRB. Fibers from DRB interact with the protein matrix in LFY, leading to a firmer structure. Since DRB is incorporated into the LFY, its components integrate with the casein proteins, promoting a more interconnected network. This enhanced network increases the structural integrity of the yogurt, resulting in a harder texture. Additionally, the natural absorbency of the rice bran fibers may reduce the free water content in the yogurt, further contributing to the increased hardness (Sairam, Gopala Krishna, and Urooj 2011). These results are in the same trend with Šmídová and Rysová (2022); Mohammadi et al. (2022). On the other side, these results were not in agreement with Demirci et al. (2017) who stated that the addition of full‐fat rice bran decreased the hardness, and the difference in results may be attributed to that full fat contains high percentage of fat which plays an important role in decreasing the hardness.

Regarding storage time, it is clear in all treatments (control and DRB‐added samples) that hardness decreased as storage time progressed which can be due to several biochemical and microbial processes that evolve during the storage period. As yogurt ages, the ongoing activity of lactic acid bacteria continues to produce acid, gradually lowering the pH. This increased acidity impacts the protein structure within the yogurt, specifically the casein proteins, causing them to further denature and potentially leading to a softer texture as the network becomes less tight and more fragmented (Ahmed et al. 2021; Alsaleem et al. 2023; Hamdy et al. 2020).

### 3.3 Viscosity

The viscosity of low‐fat yogurt (LFY) is a significant quality attribute that influences the sensory experience and overall acceptability of the product. Viscosity is affected by the formulation and the duration of storage (Kim et al. 2020). Table 3 shows that the different concentrations of DRB (treatments), the different storage periods (storage) at 4°C, and the interaction between them (treatments × storage) significantly (*p* < 0.05) affected viscosity values of LFY during 21 days of storage time.

Table 3 indicates that the addition of DRB significantly (*p* < 0.05) increased the viscosity of LFY samples compared to the control, which lacked DRB. Specifically, LFY4, which contained 2% DRB, recorded the highest viscosity values at all storage periods, starting from 2532.28 cp at 0 day to 2675.05 cp at the end of storage. This could be due to the high fiber content in DRB (Abdul‐Hamid and Luan 2000) which interacts with the protein matrix in yogurt to enhance its viscosity. The fibers from DRB create a more cohesive network by binding with casein proteins, leading to increased thickness and stability of the yogurt (Wu et al. 2023). These results were in agreement with those obtained by Rahbaran, Aarabi, and Pourabedin (2019).

Over the storage period, all LFY samples, including the control, exhibited an increase in viscosity, which was statistically significant (*p* < 0.05). For instance, the control sample's viscosity increased from 1654.80 cp on Day 0 to 1805.25 cp by Day 21. This increase can be attributed to ongoing biochemical interactions and water‐binding capabilities of the yogurt matrix, which enhance viscosity over time (Alsaleem and Hamouda 2024). The LFY samples with DRB showed a more pronounced increase, suggesting that DRB not only initially boosts viscosity but also contributes to its stability during storage.

### 3.4 Syneresis

Syneresis is an important quality attribute in yogurt as it affects both the visual appeal and textural consistency of the product. Reducing syneresis enhances consumer acceptability by maintaining a smooth, homogeneous texture and preventing the separation of whey (Temesgen and Yetneberk 2015). Statistically, Table 3 shows that the different concentrations of DRB (treatments), the different storage periods (storage) at 4°C, and the interaction between them (treatments × storage) significantly (*p* < 0.05) affected syneresis values of LFY during 21 days of storage time.

The addition of DRB significantly impacted the syneresis of low‐fat yogurt (LFY) samples over the storage period, as illustrated in Table 3. The control sample, without DRB, exhibited the highest syneresis values, increasing from 39.00% at Day 0% to 52.00% by the end of storage. In contrast, LFY samples with DRB showed notably lower syneresis, with LFY4 (2% DRB) gaining the most significant reduction, starting at 25.00% on Day 0 and reaching 33.00% by Day 21. These results suggested that adding DRB can improve the yogurt stability, which was in accordance with the previous study (Demirci et al. 2017). It was reported that dietary fibers in rice bran, such as β‐glucan, pectin, hemicellulose, and arabinogalactan, might increase water retention of yogurt, thus decreasing syneresis of yogurt (Woraratphoka et al. 2022; Wu et al. 2023).

During the storage period, syneresis increased in all samples, but the increase was less pronounced in DRB‐added samples. For instance, LFY4 showed only an 8% increase from Day 0 to 21, compared to a 13% increase in the control. The progressive increase in syneresis over time can be due to the ongoing changes in the yogurt's protein network because of acidity progress and the migration of water molecules (Alsaleem and Hamouda 2024). It was reported that lactic acid bacteria fermentation could damage the ordered crystal structure and change the monosaccharide composition of dietary fiber in rice bran (Li et al. 2022).

### 3.5 Color Characteristics

The color characteristics of yogurt are essential as they impact consumer appeal and can indicate product quality and freshness (Hutchings 2012). The obtained results in Table 3 showed that the different concentrations of DRB (treatments) significantly (*p* < 0.05) impacted the color characteristics (L*, a*, b*, and BI) of LFY samples. The addition of DRB led to remarkable changes in color parameters compared to the control sample. Specifically, LFY4 (2% DRB) gained the highest significant color changes, with L* values decreasing and a* and b* values increasing, indicating a darker and more intense color and it was expected due to the brown color of DRB, which influences the overall appearance of the yogurt. The browning index (BI) also increased significantly (*p* < 0.05) with higher DRB concentrations, with LFY4 reaching 198.01% at 0 day. However, the storage period (0, 7, 14, and 21 days) at 4°C and the interaction between treatments and storage did not significantly (*p* > 0.05) affect the color characteristics. This suggests that the primary factor influencing color changes in LFY samples is the concentration of DRB, rather than the storage duration. Similar results were obtained by Woraratphoka et al. (2022) who stated that the addition of Roseberry bran fibers in yogurt tended to decrease L* value.

### 3.6 Rheological Characteristics

**FIGURE 2:**
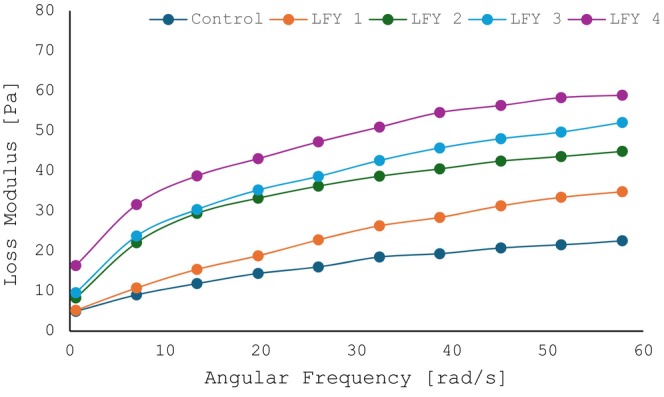
Loss modulus (Pa) of LFY samples at fresh time.

**FIGURE 3:**
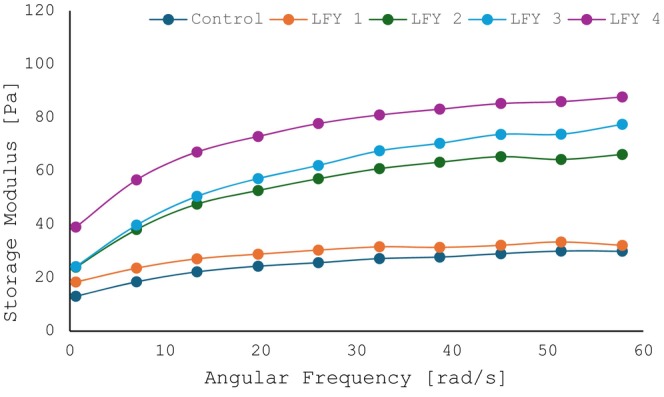
Storage modulus (Pa) of LFY samples at fresh time.

Rheology is the scientific examination of how materials flow and deform. The storage modulus (G′), loss modulus (G″), and viscosity are commonly used parameters to describe the viscoelastic behavior of materials such as yogurt. Figures 2 and 3 show that control sample gained the lowest values of G′ and G″. On the other hand, the addition of DRB to LFY samples resulted in increased values of G′ and G″ indicating a more solid‐like behavior. This is consistent with the hardness and viscosity results that further characterize this behavior, suggesting improved textural properties. This enhancement in G′ and G″ is likely due to the interaction between the DRB and the yogurt matrix, which reinforces the yogurt's structure.

### 3.7 Microbiological Analysis

Table 4 indicates that the different concentrations of DRB (treatments), the various storage periods at 4°C (storage), and the interaction between them (treatments × storage) significantly (*p* < 0.05) influenced the total bacterial counts, *Lactobacillus delbrueckii* ssp. *bulgaricus* counts, *Streptococcus thermophilus* counts, and *Bifidobacterium bifidum* counts in low‐fat yogurt (LFY) over the 21‐day storage period.

**TABLE 4:** Microbiological analysis of low‐fat yoghurt samples with different concentrations of defatted rice bran during 21‐day storage at 4°C.

Treatments^a^	Storage^b^	Total bacterial counts (log CFU/g)	*Lactobacillus dlebreuckii* ssp. *Bulgaricus* (log CFU/g)	*Streptococcus thermophilus counts* (log CFU)	*Bifidobacterium bifidum* counts (log CFU)
Control	0	7.32 ± 0.04	5.18 ± 0.04	6.56 ± 0.02	3.50 ± 0.09
	7	7.78 ± 0.14	6.42 ± 0.07	6.79 ± 0.05	3.63 ± 0.04
	14	7.84 ± 0.00	6.12 ± 0.01	6.23 ± 0.04	3.85 ± 0.07
	21	6.94 ± 0.17	5.07 ± 0.03	6.00 ± 0.08	3.38 ± 0.06
LFY 1	0	7.84 ± 0.09	7.94 ± 0.02	7.89 ± 0.02	5.56 ± 0.04
	7	7.91 ± 0.16	8.17 ± 0.05	8.33 ± 0.04	5.87 ± 0.02
	14	8.01 ± 0.01	7.79 ± 0.01	8.21 ± 0.07	5.97 ± 0.07
	21	7.62 ± 0.04	7.82 ± 0.03	7.63 ± 0.02	5.31 ± 0.05
LFY 2	0	7.94 ± 0.09	8.18 ± 0.06	7.98 ± 0.06	6.50 ± 0.03
	7	8.10 ± 0.04	8.30 ± 0.04	8.40 ± 0.08	6.75 ± 0.01
	14	8.30 ± 0.12	8.14 ± 0.01	8.21 ± 0.03	6.94 ± 0.05
	21	7.74 ± 0.07	8.09 ± 0.06	7.67 ± 0.09	6.43 ± 0.06
LFY 3	0	8.10 ± 0.08	8.40 ± 0.09	8.38 ± 0.02	7.41 ± 0.05
	7	8.21 ± 0.13	8.55 ± 0.04	8.65 ± 0.07	7.64 ± 0.06
	14	8.43 ± 0.12	8.47 ± 0.02	8.52 ± 0.05	7.81 ± 0.04
	21	7.61 ± 0.15	8.21 ± 0.07	8.24 ± 0.06	7.22 ± 0.06
LFY 4	0	6.51 ± 0.08	8.50 ± 0.03	8.64 ± 0.04	6.83 ± 0.04
	7	6.70 ± 0.03	8.78 ± 0.08	8.90 ± 0.09	6.91 ± 0.02
	14	6.81 ± 0.09	8.69 ± 0.03	8.83 ± 0.03	7.13 ± 0.00
	21	6.35 ± 0.17	8.35 ± 0.00	8.50 ± 0.07	6.71 ± 0.09
Treatments		(< 0.05)*	(< 0.05)*	(< 0.05)*	(< 0.05)*
Storage		(< 0.05)*	(< 0.05)*	(< 0.05)*	(< 0.05)*
(Treatments × Storage)		(< 0.05)*	(< 0.05)*	(< 0.05)*	(< 0.05)*

^a^ Treatments: control sample (low‐fat yogurt made without the addition of DRB), LFY1 = (low‐fat yogurt made with the addition of 0.50% DRB), LFY2 = (low‐fat yogurt made with the addition of 1% DRB), LFY3 = (low‐fat yogurt made with the addition of 1.5% DRB), and LFY4 = (low‐fat yogurt made with the addition of 2% DRB).

^b^ Storage: 0, 7, 14, and 21 days of storage at 4°C.

* Statistically significant at *p* < 0.05.

The TBC in LFY samples supplemented with DRB varied significantly across treatments and storage periods. Initially, all DRB‐added samples showed higher bacterial counts compared to the control, with LFY3 (1.5% DRB) and LFY4 (2% DRB) recording the highest values at 8.10 and 6.51 log CFU/g, respectively. Over the 21‐day storage period, TBC remained relatively stable for the DRB‐added samples, particularly LFY2 and LFY3, which maintained higher counts compared to the control. The control sample showed a noticeable decline by Day 21 (6.94 log CFU/g).

*Lactobacillus delbrueckii* *ssp.* *bulgaricus* counts were consistently higher in DRB‐added samples compared to the control. On Day 0, LFY4 exhibited the highest count (8.50 log CFU/g). This trend continued throughout the storage period, with LFY3 and LFY4 showing significantly higher counts at Day 21 (8.21 and 8.35 log CFU/g, respectively) compared to the control (5.07 log CFU/g). Similarly, the counts of *Streptococcus thermophilus* were higher in DRB‐added samples throughout the storage period. Initially, LFY4 had the highest count (8.64 log CFU/g), and by Day 21, LFY3 maintained a higher count (8.24 log CFU/g) compared to the control (6.00 log CFU/g). The Bifidobacterium *bifidum* counts followed a similar trend, with DRB‐added samples showing higher counts compared to the control. LFY4 exhibited the highest initial count (6.83 log CFU/g), and this trend persisted through Day 21, with LFY3 showing the highest count (7.22 log CFU/g) compared to the control (3.38 log CFU/g).

The increase in *Lactobacillus delbrueckii* ssp. *bulgaricus*, *Streptococcus thermophilus*, and *Bifidobacterium bifidum* counts in LFY samples supplemented with DRB can be attributed to DRB providing essential nutrients and a favorable environment that supports the growth and activity of these beneficial bacteria; therefore, it can serve as a prebiotic (Sawangwan, Kajadman, and Kulchananimit 2024). Additionally, DRB contains dietary fibers, vitamins, and minerals that enhance bacterial metabolism and proliferation. The complex carbohydrates in DRB can be fermented by these bacteria, leading to increased growth and stability (Komiyama et al. 2011).

The decrease in TBC, *Lactobacillus delbrueckii* ssp. *bulgaricus*, *Streptococcus thermophilus*, and *Bifidobacterium bifidum* counts as storage time advanced can be attributed to several factors. During storage, the yogurt environment can become less favorable for bacterial survival and proliferation due to factors such as pH changes, nutrient depletion, and the accumulation of metabolic by‐products. As the bacteria continue to metabolize the available nutrients, they produce lactic acid, which lowers the pH of the yogurt. This increased acidity can inhibit bacterial growth and reduce their viability (Ahmed et al. 2021; Alsaleem et al. 2023; Hamdy et al. 2020; Moneeb et al. 2022). Additionally, the depletion of essential nutrients over the storage period can limit bacterial activity and lead to a gradual decline in their counts (Ahmed, Hamdy, and Hammam 2020).

### 3.8 Sensory Characteristics

Table 5 depicts that the different concentrations of DRB (treatments) and different storage periods (time) at 4°C and the interaction between them (treatments × time) had a significant (*p* < 0.05) impact on all sensory characteristics of LFY samples supplemented with DRB.

**TABLE 5:** Sensory analysis of LFY samples supplemented with different concentrations of DRB during 21‐day storage at 4°C.

Treatments^a^	Storage^b^	Color and appearance (15)	Boady and texture (35)	Flavor (50)	Total (100)
Control	0	14.50	25.00	36.40	75.90
	14	14.00	22.00	40.80	76.80
	21	13.80	15.00	34.46	63.26
LFY 1	0	11.00	28.00	41.86	80.86
	14	11.00	26.00	44.52	81.52
	21	10.00	21.00	37.10	68.10
LFY 2	0	9.50	30.80	42.90	83.20
	14	8.80	28.90	45.90	83.60
	21	8.00	23.10	40.25	71.35
LFY 3	0	7.60	32.40	30.40	70.40
	14	7.00	31.00	32.10	70.10
	21	7.00	27.00	28.80	62.80
LFY 4	0	6.88	21.15	25.15	53.18
	14	6.00	18.80	27.65	52.45
	21	6.00	16.98	21.10	44.08
Treatments		(< 0.05)*	(< 0.05)*	(< 0.05)*	(< 0.05)*
Storage		(< 0.05)*	(< 0.05)*	(< 0.05)*	(< 0.05)*
(Treatments × Storage)		(< 0.05)*	(< 0.05)*	(< 0.05)*	(< 0.05)*

^a^ Treatments: control sample (low‐fat yogurt made without the addition of DRB), LFY1 = (low‐fat yogurt made with the addition of 0.50% DRB), LFY2 = (low‐fat yogurt made with the addition of 1% DRB), LFY3 = (low‐fat yogurt made with the addition of 1.5% DRB), and LFY4 = (low‐fat yogurt made with the addition of 2% DRB).

^b^ Storage: 0, 7, 14, and 21 days of storage at 4°C.

The color and appearance scores for all samples decreased over the storage period. The control sample started with the highest score (14.50) on Day 0, then slightly decreased to 13.80 by day 21. Among the DRB‐added samples, LFY 1 and LFY 2 showed higher initial scores of 11.00 and 9.50, respectively, compared to LFY 3 and LFY 4. However, LFY 4 had the lowest scores throughout the storage period, dropping from 6.88 on Day 0 to 6.00 on Day 21. This trend suggests that higher concentrations of DRB negatively affect the visual appeal of the yogurt, potentially due to the inherent color of DRB impacting the overall appearance of the product. These results agreed with those obtained by Šmídová and Rysová (2022).

The body and texture of the samples showed significant variations. Initially, LFY 3 had the highest score (32.40) at Day 0, followed by LFY 2 (30.80), indicating a positive impact of moderate DRB addition on texture. However, the scores for all samples, including the control, declined over time, noticeable decrease was observed in LFY 4, from 21.15 on Day 0 to 16.98 on Day 21. This suggests that while DRB can initially improve the texture of LFY, higher concentrations of DRB lead to more negative influences on the texture of LFY. These results agreed with those obtained by Doan et al. (2021).

Flavor scores were highest in LFY 2, starting at 42.90 and maintaining rather high scores throughout the storage period, with a score of 40.25 on Day 21. The control sample also showed good flavor scores during the storage period, despite a significant drop by Day 21. LFY 1 and LFY 3 had irregular scores throughout the storage period. LFY 4 consistently had the lowest flavor scores, starting at 25.15 and lowering to 21.10 by Day 21, indicating that higher DRB levels might negatively impact the flavor score. This observation is consistent with Mishra (2017).

Total scores, representing overall acceptability, were highest for LFY 2, indicating that a 1% addition of DRB is optimal for balancing the sensory characteristics of color, texture, and flavor. LFY 1 also showed good overall acceptability, while LFY 3 and LFY 4 had lower scores, with LFY 4 being the least acceptable. The control sample maintained good acceptability but showed a noticeable decline by Day 21.

## 4 Conclusion

In conclusion, adding DRB to low‐fat yogurt (LFY) significantly improved its nutritional value and probiotic content. The inclusion of DRB increased total solids, protein, ash, fiber, and antioxidant activity, while also enhancing the yogurt's hardness and viscosity. DRB‐enriched yogurt samples maintained higher levels of beneficial bacteria, such as *Lactobacillus delbrueckii* ssp. *bulgaricus*, *Streptococcus thermophilus*, and *Bifidobacterium bifidum*, throughout the 21‐day storage period, highlighting DRB's prebiotic benefits. However, higher concentrations of DRB negatively impacted the yogurt's color and sensory qualities, with the 2% DRB addition receiving the lowest sensory scores. The best balance of improved nutrition and acceptable taste was found with up to 1.5% DRB, making it an excellent addition for creating healthier, functional yogurt products.

## Author Contributions

**Khalid A. Alsaleem:** data curation (equal), formal analysis (equal), funding acquisition (equal), investigation (equal), methodology (equal), project administration (equal), resources (equal), software (equal), supervision (equal), validation (equal), visualization (equal), writing – review and editing (equal). **Mahmoud E. A. Hamouda:** conceptualization (equal), data curation (equal), formal analysis (equal), investigation (equal), methodology (equal), project administration (equal), resources (equal), software (equal), supervision (equal), validation (equal), visualization (equal), writing – original draft (equal), writing – review and editing (equal).

## Data Availability

Data are contained within the article.
